# Bioprospecting of a Native Plant Growth-Promoting Bacterium *Bacillus cereus* B6 for Enhancing Uranium Accumulation by Sudan Grass (*Sorghum sudanense* (Piper) Stapf)

**DOI:** 10.3390/biology14010058

**Published:** 2025-01-13

**Authors:** Longyuan Wu, Lijuan Zhang, Ning Wang, Wei Huang, Yanzhi Wang, Meng Sun, Guofeng Zheng, Wei Wang, Chong Shi

**Affiliations:** 1 College of Resources and Environment, Xinjiang Agricultural University, Urumqi 830052, China; wulongyuan21@163.com (L.W.); wang58462@163.com (Y.W.); sunmeng011216@163.com (M.S.); 2 Institute of Applied Microbiology, Xinjiang Academy of Agricultural Sciences/Xinjiang Laboratory Special Environmental Microbiology, Urumqi 830091, China; zhanglijuan@xaas.ac.cn (L.Z.); wangning950203@163.com (N.W.); ryan_huangwei1991@126.com (W.H.); 3 College of Grassland Science, Xinjiang Agricultural University, Urumqi 830091, China; 4 Nuclear and Radiation Safety Center, Beijing 100082, China; zhenggf163_lzu@163.com

**Keywords:** *Bacillus cereus*, plant growth-promoting activity, uranium stress

## Abstract

Phytoremediation technology is viewed as a potential solution for addressing soil uranium contamination. Sudan grass (*Sorghum sudanense* (Piper) Stapf.), noted for its robust root structure and resilience to heavy metals, has garnered significant attention. This paper investigates a strain of uranium-tolerant bacterium, B6, obtained from the inter-root environment of native plants in soil contaminated with uranium tailings. The bacterium was identified as Bacillus cereus. Genomic analyses and assessment of uranium tolerance-promoting properties showed that strain B6 not only exhibited high uranium tolerance, but also possessed beneficial properties such as phosphorus solubilization and iron-producing carriers. In this study, we used strain B6 as an inoculant in combination with Sudan grass for germination and potting experiments. The findings demonstrated that Bacillus cereus B6 could substantially mitigate the adverse effects of uranium stress on Sudan grass, boost the plant’s antioxidant response, significantly increase the root length and dry biomass of Sudan grass, and facilitate the accumulation of uranium in the roots, as well as its translocation to the aboveground portions. The study showed that PGPB strain B6 can significantly enhance the effect of plant accumulation of uranium and increase the potential of Sudan grass to become a uranium-rich plant, which provides an important scientific basis and application prospect for the use of microbial-assisted Sudan grass remediation technology to treat uranium-contaminated soil.

## Simple Summary

Remediation of uranium-contaminated soils is a crucial and arduous task essential for safeguarding environmental protection and human health. In this study, a bacterium named *Bacillus cereus* B6 was isolated from the rhizosphere of native plants thriving in uranium tailing-contaminated soil. This bacterium exhibits high tolerance to elevated concentrations of uranium and enhances plant growth by facilitating nutrient uptake. Seed germination and potting experiments were conducted under simulated uranium contamination conditions, with B6 inoculated onto *Sorghum sudanense*. The results demonstrated that B6 significantly improved the efficiency of uranium uptake by Sudan grass and promoted the translocation of uranium to the aboveground portions of the plant. The research confirms the substantial effectiveness and favorable future of integrating *Sorghum sudanense* with *Bacillus cereus* for the purification of soils polluted with uranium, establishing a theoretical basis for its practical use.

## 1. Introduction

Uranium contamination presents a significant threat to global health. Humans are primarily exposed to uranium over a prolonged period through the ingestion of contaminated water, food, and soil particles [1,2]. This exposure can result in a multitude of adverse health outcomes, ranging from lung and bone cancers to kidney damage, hypertension, autoimmune disorders, and reproductive issues [2,3]. In natural settings, uranium is predominantly found in two forms: tetravalent uranium (U(IV)) and hexavalent uranium (U(VI)). Notably, U(VI) exhibits significantly higher mobility and bioavailability compared to other oxidation states, resulting in more pronounced toxicity characteristics [4]. Soil contamination with uranium can originate from both natural processes and anthropogenic activities. Uranium mining is one of the most frequently reported sources, where the waste left behind contains elevated levels of uranium and other heavy metals, thereby contaminating soil, air, and water [5,6]. The migration of these pollutants in the environment increases the number of uranium hotspots near mining areas, amplifying public health risks [7,8]. As a heavy metal with both radiological and chemical toxicity, uranium poses a long-term threat to the environment, lasting for millennia once it enters the soil.

There are various methods for cleaning up environments contaminated with uranium [9]. Phytoremediation of soils stands out as a sustainable solution due to its eco-friendliness, low cost, and effectiveness [10,11]. Several plant species have been reported to exhibit tolerance and bioaccumulation capacity for uranium, including *Lolium perenne* L., *Helianthus annuus* L., *Brassica juncea*, and *Macleaya cordata* [12,13,14].

However, the efficiency of phytoremediation on its own is frequently constrained by the gradual growth rate of plants, which can be affected by multiple stressors such as temperature fluctuations, salinity levels, pH conditions, soil fertility, and the extent of contamination [15,16,17]. Integrating plant growth-promoting rhizobacteria (PGPR) into the phytoremediation system for heavy metal-contaminated soils can markedly boost the remediation processes [18,19].

Phytoremediation facilitated by microbes is a well-studied strategy for tackling soil heavy metal contamination [20]. PGPR can boost plant growth and enhance their resistance to heavy metal (HM) stress through various mechanisms, both direct and indirect. These include the solubilization of phosphorus [21], the fixation of atmospheric nitrogen [22], the synthesis of siderophores [23], the production of phytohormones [24], and the generation of antibiotics and lytic enzymes, among other functions. In environments polluted with heavy metals, PGPR help regulate the activity of plant antioxidants and neutralize reactive oxygen species (ROS) generated by these metals [25], thereby reducing oxidative stress in plants. Moreover, PGPR release low molecular weight organic acids and metal-specific chelators [26,27], which facilitate the desorption of metal ions from soil particles and increase the bioavailability of heavy metals. Studies have shown that indigenous PGPR can boost plant resilience to heavy metals and stimulate their growth through synergistic interactions [22]. While the use of PGPR in phytoremediation has been extensively explored for various heavy metal pollutants, there is still a limited amount of research dedicated to its application in remediating uranium-contaminated sites [18,25,28].

A promising strategy for enhancing phytoremediation involves utilizing bioenergy crops with high biomass production potential, such as *Sorghum sudanense* [29], *Miscanthus* [30], *Vetiver zizanioides*, and *Cannabis sativa* [31]. *Sorghum sudanense* (Piper) Stapf., an annual pasture grass, is noted for its large biomass, rapid growth rate, and strong tolerance to pollutants like polycyclic aromatic hydrocarbons (PAHs) and heavy metals [32,33,34]. Notably, it can achieve substantial biomass even in the presence of heavy metals [35,36]. Although studies on the interaction between Sudan grass and uranium are limited compared to other heavy metals, it has been shown that Sudan grass is tolerant to certain concentrations of uranium and has the potential to become a hyperaccumulator [36,37]. A number of studies have explored improving phytoremediation efficiency by introducing exogenous remediation agents to mitigate the effects of uranium stress on Sudan grass by increasing Sudan grass biomass, enhancing Sudan grass antioxidant capacity [34,38,39], and introducing substances such as chelating agents, surfactants, and others. These additives aim to improve soil nutrients and promote the growth of Sudan grass. Notably, one study used PGPR with Sudan grass for this purpose [11]. Despite these efforts, the use of *Sudanese sorghum* for microbial-assisted remediation of soils contaminated with heavy metals, especially uranium, is still under-explored compared to other approaches. Therefore, it is crucial to further investigate the potential of PGPR in combination with bioenergy crops such as Sudan grass to develop more effective remediation strategies for contaminated soils

In the previous study, we isolated a strain of uranium-tolerant PGPR from the inter-roots of native plants growing naturally in a uranium mine tailings pond. We investigated the uranium-tolerant and growth-promoting ability of strain B6 and its effect on physiological and uranium enrichment indicators of the uranium-enriched plant *Sorghum sudanense* under uranium stress through genomic characterization, uranium-tolerance and growth-promoting characterization, as well as seed germination and potting tests. The primary objective of this study was to investigate the potential of the uranium-tolerant probiotic B6 and its ability to assist in the combined remediation of uranium contamination using *Sorghum sudanense*. In addition, it aims to provide a scientific basis for further exploring the feasibility of microbial-assisted Sudan grass remediation techniques for remediating uranium-contaminated soils.

## 2. Materials and Methods

### 2.1. Plant and Bacteria for Phytoremediation

*Sorghum sudanense* (Piper) Stapf was used in an in vivo phytoremediation experiment. The seeds were sourced from local markets in Shaoguan, Guangdong, China. The uranium-resistant bacterium strain B6 was isolated from rhizosphere soils collected from the tailings dam of a uranium mine located in Shaoguan City, Guangdong Province, China (25°18′12″ N, 113°53′57″ E) following the procedure of Pranaw et al. [21] in April of the year 2022. The half maximal inhibitory concentration (IC50) was determined using experiments conducted in Nutrient Broth (NB) medium. Uranyl nitrate hexahydrate (UO_2_(NO_3_)_2_·6H_2_O) was used to simulate uranium stress in the environment. All isolates were cultured in NB spiked with U (VI): 0~800 mg·L^−1^, cultured at 30 °C for 48 h with shaking at 180 rpm. Bacterial growth was measured every two hours on a UV–visible (UV-Vis) spectrophotometer at 600 nm [40]. The same method is used for the other heavy metals cadmium (Cd(NO_3_)_2_), chromium (K_2_Cr_2_O_7_), lead (Pb(NO_3_)_2_), manganese (MnCl_2_), and zinc (Zn(NO_3_)_2_).

### 2.2. Whole-Genome Sequencing and Identification of Uranium-Resistant Strain B6

Genomic DNA from strain B6 was extracted using a commercial kit provided by Omega Bio-tek. The purified genomic DNA was then sequenced using Single Molecule, Real-Time (SMRT) technology at Beijing Novogene Bioinformatics Technology Co., Ltd. (Beijing, China) Low-quality reads were filtered using SMRT Link v8.0, and the high-quality reads were assembled into a single, gapless contig using Canu version 2.0 [41]. Genome sequences were submitted to WGS database of NCBI with accession No. CP133649-CP133652 and annotated by the NCBI Prokaryotic Genome Annotation Pipeline (PGAP). A circular genome map was drawn using Bakta Web tool (Software: 1.9.1|DB: 5.0.0) [42]. The genome sequences generated in this study have been submitted to the NCBI Genome Archive and are accessible under BioProject ID PRJNA1011042.

The taxonomic characterization proceeded by searching the 16S rRNA genes against the EzTaxon database (https://www.ezbiocloud.net), accessed on 23 August 2023 [43] and further assessed by analyzing both average nucleotide identity (OrthoANI) [44] and the digital DNA-DNA hybridization (dDDH) [45] values between strain B6 and its associated strains. The genes corresponding to plant-growth promotion and heavy metal resistance were prospected.

### 2.3. In Vitro Assessment of Plant Growth-Promoting (PGP) Properties

Inoculate into 50 mL of NB medium and shake at 30 °C for 24 h as seed solution, and take 5 μL of the bacterial solution to the screening medium of each growth-promoting feature respectively.

The ability to solubilize phosphates was assessed on National Botanical Research Institute Phosphate (NBRIP) agar plates, where insoluble Ca_3_(PO_4_)_2_ served as the sole phosphorus source [45]. Similarly, potassium solubilization was evaluated on Aleksandrov solid medium plates, using insoluble mica (potassium aluminum silicate) as the exclusive potassium source [46]. Plates were incubated at 28 °C for 5–7 days, and the presence of a clear halo around the colonies indicated successful P or K solubilization [47]. Indole acetic acid (3-indole acetic acid, IAA) production was determined by Salkowski’s colorimetric assay [48].

The production of siderophores was analyzed using Chromo-Azurol-S (CAS) agar [49]. Plates were incubated at 28 °C for 5–7 days, and the appearance of a halo around the colonies indicated siderophore production. N_2_ fixation was assayed on nitrogen-free medium (NBF) [50]. Organic acid production was analyzed on Nutrient Agar (NA) plates containing bromocresol purple [51] with the ability to produce organic acids.

Qualitative determination of enzyme degradation activity: protease activity was tested on plates containing casein [52] and cellulase activity on plates containing carboxymethylcellulose (CMC) [53]. All PGPB activities were analyzed in triplicate.

### 2.4. Effect of Inoculation on Seed Germination

To prepare the inoculum, strain B6 was cultivated overnight in Nutrient Broth (NB) at 28 °C with agitation at 200 rpm. The cells were subsequently collected via centrifugation at 10,000× *g* for 10 min, rinsed twice with sterile distilled water, and ultimately resuspended in sterile distilled water to attain an OD_600_ of about 1.0, suitable for subsequent inoculation procedures.

Sudan grass seeds underwent surface sterilization by being immersed in a 1% (mass/volume) sodium hypochlorite solution for a duration of 10 min, with occasional gentle agitation, followed by thorough rinsing using sterile distilled water [54]. For the inoculation process, the seeds, now sterilized, were immersed in the prepared bacterial suspension for a period of 2 h at ambient temperature. The control group received only sterile water in place of the bacterial solution. Subsequently, the seeds were placed into petri dishes, each containing 20 seeds, on double-layered filter paper using sterile forceps and incubated in the dark at a temperature of 25 °C for a week. The filter paper was initially moistened with 10 mL of uranium solutions at concentrations of 0, 50, and 100 mg·L^−1^, respectively. After that, germination was counted every day, and on the 3rd day, germination potential was calculated, and on the 7th day, germination percentage, root crown length, and fresh weight were calculated. Three replicates of each treatment were used in this experiment. Calculation formulae refer to Feizi et al. [55].

(1) $$Germinative\;energy\;\left(GE\right)\%=\left(\frac{seeds\;germinated\;after\;3\;days}{total\;no.\;of\;seeds}\right)\times 100\%$$

(2) $$Germination\;percentage\;(GP)\;\%=(\frac{seeds\;germinated\;after\;7\;days}{total\;no.\;of\;seeds})\times 100\%$$

(3) $$Germination\;index\;(GI)=\sum \frac{Gt}{Dt}$$

where *Gt* refers to the number of seed germination on *t* day, and *Dt* refers to the corresponding number of seed germination days.

(4) Vital index (VI)=GI×Lr

where *Lr* refers to the average root length.

### 2.5. Cultivation of Plants Under Greenhouse Conditions

A pot experiment was conducted in a greenhouse, located at Xinjiang Academy of Agricultural Sciences, China, in October 2023. The soils used in the pots were collected from a farmland 20 km away from the uranium mine, homogenized through 5 mm sieve, and thoroughly mixed with uranyl nitrate to simulate uranium-contaminated soil. After full mixing, the soil was kept in a cool place for 8 weeks and balanced, then divided into φ29 cm × 35 cm flowerpots with 2.5 kg of soil in each pot. Soil physicochemical properties are shown in Table 1.

The seeds were subjected to a surface sterilization process as previously detailed. Following sterilization, the seeds were allowed to pre-germinate in sterile petri dishes lined with dampened filter papers at a temperature of 25 °C for a period of 48 h. Once germination occurred and seeds reached a uniform developmental phase, they were initially transplanted into seedbeds for a duration of one week. Subsequently, they were moved into pots, with each pot accommodating 10 plants and filled with 2.5 kg of artificially contaminated soil. For comparative purposes, pots filled with an equivalent measure of unadulterated soil served as the control group. Half of the pots were watered once a week with 300 mL tap water. The other half were watered with 300 mL of bacterial solution (10^6^ CFU mL^−1^) every week. Four different treatments, metal treatment + inoculation, metal treatment + no inoculation, original soil + inoculation, and original soil + no inoculation, were established and each treatment was replicated five times. Plants were grown in a greenhouse for 6 weeks with natural light intensity and temperature controlled at 22–35 °C.

Following 42 days of cultivation, the plants were collected, washed with tap water, and partitioned into root and shoot sections. The lengths of the leaves and roots were recorded, and the fresh biomass was assessed (with n = 30 for each treatment group). To ascertain the dry mass, a segment of the root and stem biomass was dried at 80 °C until a constant weight was reached.

### 2.6. Determination of Uranium Accumulation in Plants

Samples consisting of 0.3 g dry weight of shoots or roots (prepared as a homogeneous mixture from pieces of 30 treated plants) were subjected to digestion using 6 mL of nitric acid (HNO_3_), 0.5 mL of hydrofluoric acid (HF), and 1 mL of hydrogen peroxide (H_2_O_2_) at temperatures ranging from 120 °C to 200 °C for a duration of 1 h. The uranium concentrations were then quantified using an inductively coupled plasma mass spectrometer (ICP-MS, model X-Series II by Thermo Fisher Scientific, (Waltham, MA, USA)). By interpolating the elemental signal values obtained from the samples onto a calibration curve, the total uranium concentration within each sample was determined.

The plant concentration factor (*PCF*) was calculated on dry weight basis as per [36,56]

(5) $$PCF=C_{plant}\;/\;C_{soil}$$

where *C_plant_* and *C_soil_* represent the metal concentration in plant roots and soils on dry weight basis, respectively.

The plant transfer factor (*TF*) was also calculated on dry weight basis [56].

(6) $$TF=C_{shoot}\;/\;C_{root}$$

where *C_shoot_* and *C_root_* represent the heavy metal concentration in shoots and roots of plants on dry weight basis, respectively.

### 2.7. Measurement of Malondialdehyde (MDA) Content, Reduced Glutathione (GSH) and Oxidized Glutathione (GSSG) Content, and Antioxidant Enzyme Activity

The contents of malondialdehyde (MDA), glutathione (GSH), and oxidized glutathione (GSSG), as well as the enzyme activities of peroxidase (POD), catalase (CAT), superoxide dismutase (SOD), and polyphenol oxidase (PPO), were analyzed using a spectrophotometer (SPECORD210plus, (Analytik Jena, Jena, Germany)).

Levels of malondialdehyde (MDA), a biomarker for lipid peroxidation and oxidative stress, were evaluated using the technique reported by Du et al. [57]. In this process, 0.2 g of fresh leaf material was homogenized in 10 mL of a 10% trichloroacetic acid solution, followed by centrifugation at 12,000× *g* for 10 min. The supernatant, collected after centrifugation (2 mL), was then mixed with 2 mL of a 0.6% thiobarbituric acid solution and incubated in boiling water at 100 °C for a period of 15 min. Once the mixture had cooled, it underwent a second centrifugation at 12,000× *g* for an additional 10 min. The absorbance of the supernatant was measured at 532 nm, 600 nm, and 450 nm. The MDA concentration in the plant samples was calculated in nanomoles per gram using the formula provided by Du et al. [57].

The levels of glutathione (GSH) and its oxidized form (GSSG) were ascertained employing a spectrofluorimetric technique as detailed in reference [58]. For this, 0.5 g of leaf tissue was processed in a 4 mL mixture comprising a blend of 0.1 M potassium phosphate buffer at pH 8.0 and 25% metaphosphoric acid in a volume ratio of 3.75:1. The mixture was then centrifuged for 10 min at 8000× *g*, followed by a secondary centrifugation of the supernatant for 5 min at 13,000× *g*. The concentrations of GSH and GSSG in the supernatant were measured using the reagents and protocols outlined by Marta et al. [59]. The GSH levels were reported in micromoles per gram of fresh plant weight, while GSSG levels were noted in nanomoles per gram of fresh plant weight.

For enzyme extraction, 0.1 g of fresh leaf tissue was homogenized in a mortar with 1 mL of extraction buffer. The buffer comprised 0.1 M phosphate buffer (pH 7.5), supplemented with 0.5 mM EDTA, and the process was conducted at a temperature of 4 °C. The homogenate was then centrifuged at 4 °C for 20 min at 15,000× *g*. Following centrifugation, the supernatants were collected for subsequent analysis of enzyme activities.

The peroxidase (POD) activity was assessed based on the procedure outlined by Reuveni [60]. In this process, 50 µL of the supernatant was combined with 3 mL of the assay solution. This solution was composed of 15 mM sodium phosphate buffer at pH 6.0, 1 mM hydrogen peroxide (H_2_O_2_), and 0.1 mM o-methoxyphenol (guaiacol). Spectrophotometric monitoring of the absorbance increase at 470 nm was conducted to track the reaction. The POD activity was quantified as the rate of absorbance change per minute per gram of fresh weight.

The catalase (CAT) activity was determined following the procedure outlined by Goel and Sheoran [61]. This method required the combination of 0.6 mL enzyme extract with 2.0 mL of a 30 mM potassium phosphate buffer (pH 7.0) and 0.1 mL of a 20 mM hydrogen peroxide (H_2_O_2_) solution, which was then incubated for 1 min at a temperature of 28 °C. The degradation of H_2_O_2_ was monitored by measuring the absorbance at 240 nm. CAT activity was quantified as a reduction of 0.1 absorbance unit at 240 nm per minute.

Activity levels of superoxide dismutase (SOD) were assessed following the methodology described by Batool et al. [62]. The process began with the combination of 0.1 mL enzyme extract and a reaction mixture comprising 1.5 mL of a 50 mM sodium phosphate buffer set at pH 7.8, 0.3 mL of a 130 µM methionine solution, 0.3 mL of a 750 µM nitro-blue tetrazolium (NBT) solution, 0.3 mL of a 100 µM EDTA-Na_2_ solution, 0.3 mL of a 20 µM riboflavin solution, and 100 µL of distilled water. The mixture was exposed to light at 4000 lux for 20 min. Post-exposure, the absorbance was measured at 560 nm. SOD activity was quantified as the amount of enzyme necessary for a 50% suppression of NBT reduction.

Polyphenol oxidase (PPO) activity was determined following the guidelines set by Sharma et al. [63]. The assay’s composition included 2 mL of a 0.1 M phosphate buffer at pH 7.0, 1 mL of a 0.1 M catechol solution, and 0.5 mL of the enzyme extract. The mixture was kept at 25 °C for 5 min to allow for incubation. Subsequently, 1 mL of 2.5 N H_2_SO_4_ was introduced to terminate the enzymatic process. The absorbance, indicative of benzoquinone production, was recorded at 495 nm. PPO activity was quantified in units per gram of fresh weight (U/g FW), where each unit (U) corresponds to the enzyme’s ability to induce a 0.01 absorbance shift at 420 nm per minute within the reaction system.

### 2.8. Statistical Analysis

All experiments were conducted in triplicate, and the data presented are the mean ± standard error. Comparisons between means were performed using one-way ANOVA with SPSS v.25.0 (IBM Corp., Armonk, NY, USA). Statistical significance (*p*-value ≤ 0.05) was evaluated and indicated in tables and figures using different letters.

## 3. Results

### 3.1. Identification and Genomic Features of Strain B6

**Figure 1:**
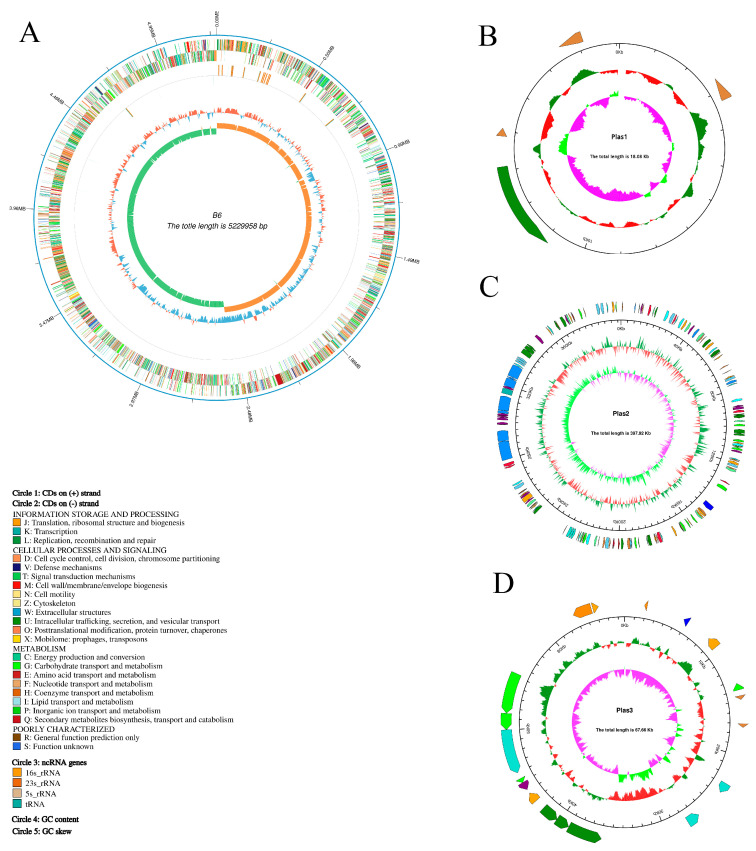

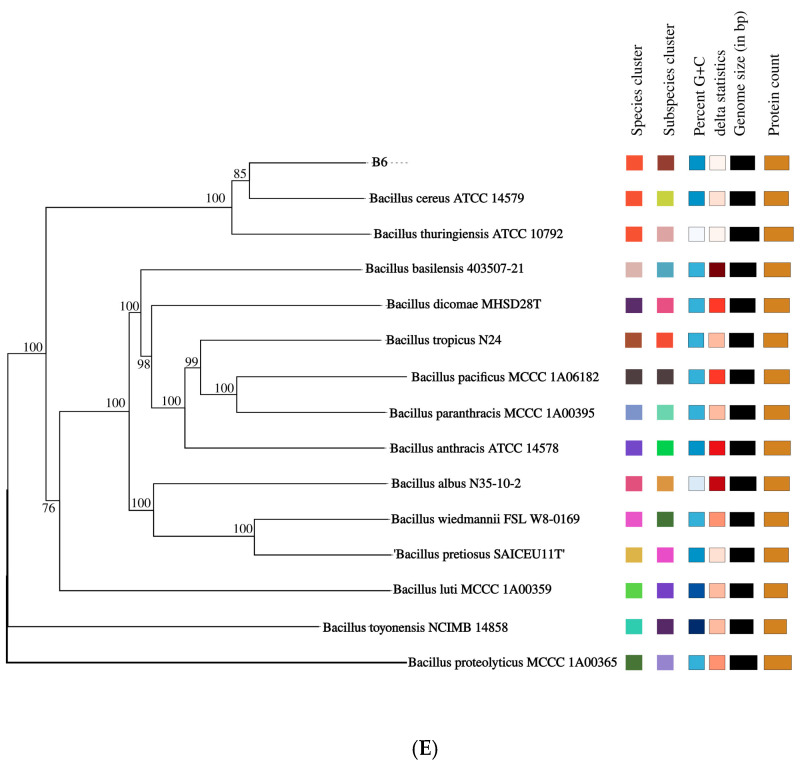
(**A**–**D**): Circos diagrams represent the genome map of the strain B6 bacterial chromosome and three plasmids, respectively, indicating the COG annotation, ncRNA, GC content, and GC skew. There was no ncRNA in the plasmid sequences. (**E**): The phylogenetic evolutionary tree was inferred using FastME 2.1.6.1 based on GBDP distances derived from genome sequences. Branch lengths are scaled according to the GBDP distance formula d5. Numbers above branches represent GBDP pseudo-bootstrap support values greater than 60% from 100 replications, with an average branch support of 96.5%. The tree was rooted at the midpoint.

The whole genome of strain B6 had a total length of 5,713,608 bp and a GC content of 35.06%. It consisted of a circular chromosome (5,229,958 bp) and three circular plasmids (18,079 bp, 397,915 bp, 67,656 bp) encoding 5909 genes, 107 tRNAs, and 42 rRNAs (Figure 1A–D). The total length of the coding region was 4,752,687 bp, which accounted for 83.18% of the whole genome, and the average length of the coding genes was 804 bp.

The 16S rRNA gene similarity of the B6 strain to the closest type strains were higher than the suggested species declination cut-off of 98.5% (Table S1). The phylogenetic tree based on the whole genome sequence of strain B6 showed that strain B6 and *Bacillus cereus* ATCC 14579 were in the same branch with the closest affinity (Figure 1E), and the ANI of both was (>97%), while dDDH was (>70%). Combined ANI and dDDH comparison results indicate B6 can be considered as a *Bacillus cereus* species (Table 2).

*B. cereus* B6 genome analysis revealed the presence of several genes that are involved in heavy metal (HM) tolerance and plant growth promotion, including nitrogen assimilation, iron sequestration, phosphate solubilization, heavy metal resistance (Table S2). The abundance of genes involved in HM tolerance and plant growth promotion in the strain B6 genome is in agreement with the functional data showing that strain B6 tolerates multiple metals, and previous reports on the other B. cereus strains which were used as inoculants for increasing HM tolerance in various crops [27,64,65].

### 3.2. Determination of the Minimal Inhibitory Concentrations (MICs) for Uranium and Metals

**Figure 2:**
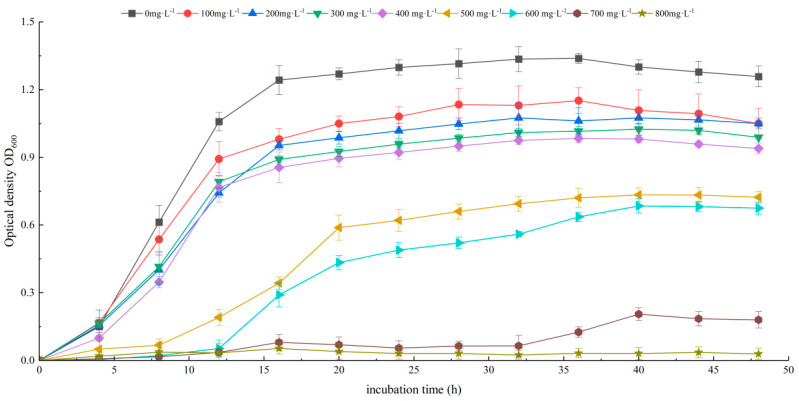
Growth curves of strain B6 in NB with uranium.

Cultured in uranium-containing NB liquid medium, the growth curve of B6 was S-shaped (Figure 2) when the concentration of uranium was lower than 400 mg·L^−1^; the growth of B6 was inhibited, the delay period increased significantly, and the number of bacteria also decreased significantly after entering the stable period when the concentration of uranium was 500 mg·L^−1^. The growth was completely inhibited when the concentration of uranium in the medium reached 700 mg·L^−1^ (Figure 2). Similarly, the resistance of the strain to other heavy metals (Cd, Cr, Pb, Mn, and Zn) was evaluated, and the minimum inhibitory concentrations (MICs) were calculated in NB liquid medium. The MIC concentrations for Cd, Cr, Pb, Mn, and Zn were 800 mg·L^−1^, 400 mg·L^−1^, 600 mg·L^−1^, 400 mg·L^−1^, and 400 mg·L^−1^, respectively (Figure S1A–E).

### 3.3. In Vitro Evaluation of the Growth-Promoting Properties of Strain B6

**Figure 3:**
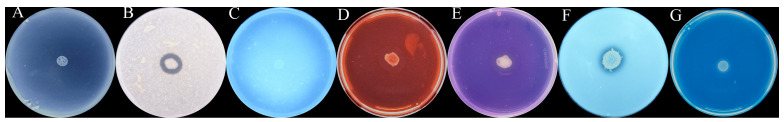
Qualitative detection of growth-promoting properties of strain B6: (**A**) phosphate solubilization, oil-like colonies (**B**) protease activity, hyaline circles; (**C**) nitrogen fixation, growth on nitrogen-free medium (**D**) cellulase activity, hyaline circles; (**E**) organic acid production, yellow hyaline circles; (**F**) phosphate solubilization, hyaline circles around the strain; (**G**) iron-carrier production capacity, hyaline circles.

The results (Figure 3) demonstrated that this strain exhibited siderophore production and possessed the abilities to solubilize phosphate and potassium, fix nitrogen, and produce organic acids. Additionally, it showed protease and cellulase activities. However, the ability to produce indole-3-acetic acid (IAA) was not detected.

### 3.4. Effects of Strain B6 on Growth and Uranium Enrichment of Sudan Grass in Uranium-Contaminated Soil

#### 3.4.1. Effects of Strain B6 on Seed Germination Under Uranium Stress

The germination experiments of seeds treated with 0, 50, and 100 mg·L^−1^ uranium concentration showed that all germination indices measured decreased both the B6 inoculation group and uninoculated control group decreased with the increase of uranium concentration. In particular, the length of the roots decreases significantly, indicating that the roots of seedlings were highly sensitive to uranium and were most affected by uranium.

Under the same cultivation conditions, seed germination index, vital index, and root length were significantly increased in plants treated with strain B6 compared to the uninoculated control (Table 3, Figure S2). The B6-treated group without uranium stress had the highest root and shoot lengths of all treatments (89.8 mm and 67.8 mm, respectively). This suggests that strain B6 can promote seed germination and alleviate the stress of uranium on seed germination.

#### 3.4.2. Effects of Strain B6 on Growth and Uranium Enrichment of Sudan Grass in Uranium-Contaminated Soil

**Figure 4:**
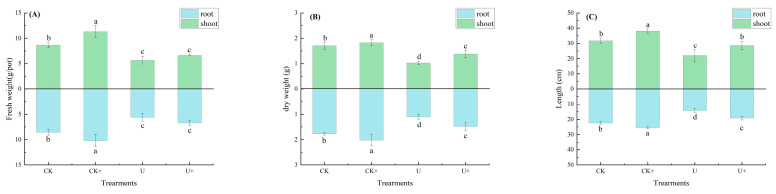
Effect of B6 inoculation on growth and uranium accumulation of Sudan grass (+: B6 treatment): (**A**) fresh weight; (**B**) dry weight; (**C**) shoot and root length. Different letters indicate significant differences (*p* < 0.05).

The greenhouse study on Sudan grass cultivation in soil with uranium contamination demonstrated that the B6 inoculant had a pronounced impact on key growth indicators: the height of the plants, the length of their roots, and the dry weight of their biomass, with the differences being statistically significant at the *p* < 0.05 level, as depicted in Figure 4A–C. In the control group without inoculation, plant height, root length, and dry and fresh weight decreased significantly under 100 mg·kg^−1^ uranium stress. The plant height decreased by 30.8%, root length decreased by 36.3%, and biomass decreased by 34% (*p* < 0.05). Growth was significantly inhibited, indicating that the high concentrations of uranium in the soil was stress toxicity to plant growth.

**Figure 5:**
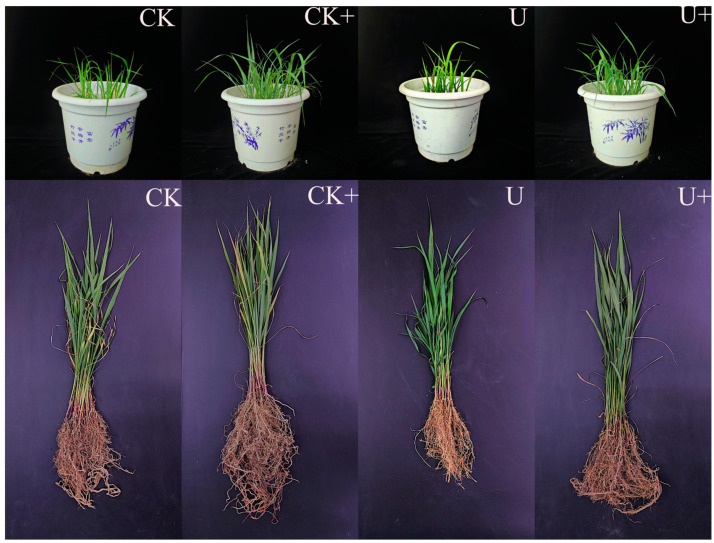
Representative plants from each treatment.

Plants that received the B6 strain inoculation displayed enhanced growth performance in comparison to their non-inoculated counterparts, as illustrated in Figure 5. Notably, under conditions of uranium stress, the application of strain B6 markedly improved plant height, root length, and dry weight. This suggests that strain B6 mitigated the growth-inhibiting effects of uranium stress and fostered plant growth even in the presence of uranium stress.

The contents of uranium in roots increased by 12% and that of the aboveground parts by 274% after strain B6 treatment (Table 4). Roots were the main part of uranium enrichment in plants, and strain B6 enhanced uranium enrichment in roots and uranium transport to the aboveground part of the plant and enhanced uranium enrichment within the plant.

### 3.5. Effect of Strain B6 on the Antioxidant Response in Plants Under Uranium Stress

**Figure 6:**
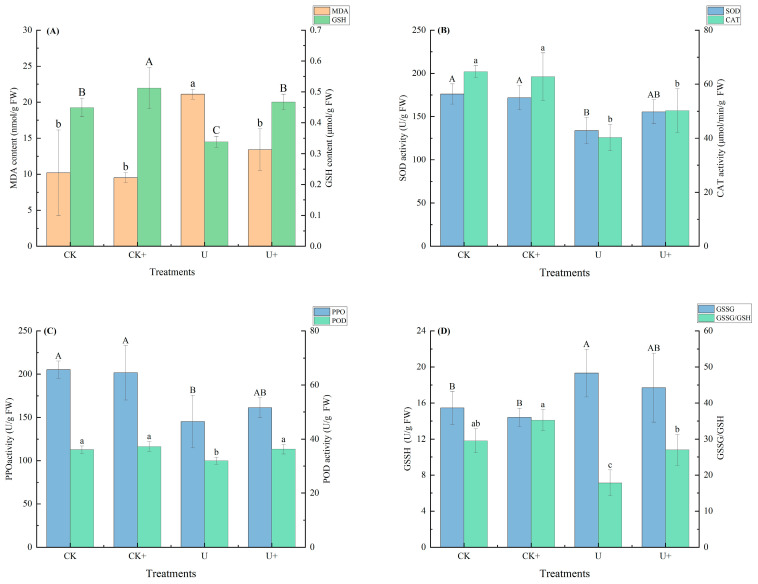
Effect of inoculation with B6 on enzyme activity of Sudan grass under uranium stress (+: B6 treatment): (**A**) MDA content; (**B**) SOD and CAT activities; (**C**) PPO and POD activities; (**D**) GSSG content and GSH/GSSG value. Different letters indicate significant differences (*p* < 0.05).

To assess the capacity of strain B6 to mitigate oxidative stress under conditions of uranium exposure, both non-enzymatic and enzymatic antioxidant defenses were scrutinized in Sudan grass cultivated in soil with elevated uranium levels. The results are shown in Figure 6A–D. The MDA content was significantly increased by 106% in U^6+^ treatment alone compared to control treatment. CAT, POD, SOD, and PPO enzyme activities were significantly reduced by 37.8%, 11.1%, 24.0%, 29.2%, and 39.6% under U^6+^ treatment alone. Exposure to uranium stress resulted in a 24.4% decrease in GSH but an additional 30% accumulation of GSSG (*p* < 0.05).

It can be seen that uranium stress inhibited the antioxidant responses of the plants, while inoculation of B6 under uranium stress resulted in a 50.33% decrease in MDA accumulation and a 38.13% increase in GSH content (*p* < 0.05) compared to uranium treatment. In addition, B6 treatment significantly increased POD and PPO enzyme activities by 12.5% and 11.0%, respectively, compared to uranium treatment alone but did not reach the corresponding values of the control without uranium and B6 treatments. B6 treatment increased non-enzymatic substance GSH content by 38.2%, decreased GSSG content by 8.3% (*p* < 0.05), and increased SOD and CAT by 24.8% and 16.2%, respectively, but with non-significant results. The results indicated that inoculation with B6 could alleviate the lipid peroxidation stress of uranium on plants, reduce MDA accumulation, and increase the antioxidant enzyme activities of plants.

## 4. Discussion

Phytoremediation technology is considered a highly promising method for managing soil uranium pollution [66,67]. Sudan grass exhibits rapid growth, large biomass, wide distribution, strong regenerative ability, and well-developed underground rhizomes and root systems, effectively reducing the loss and spread of pollutants [32,68]. This study shows that it also demonstrates high tolerance to heavy metal stresses, including Ni [32], Si [68], Pb [69], and U. Generally, the threshold for hyperaccumulation of heavy metals in plants is defined as dry weight concentrations above 100 mg/kg for cadmium (Cd); 1000 mg/kg for cobalt (Co), copper (Cu), nickel (Ni), and lead (Pb); and 10,000 mg/kg for manganese (Mn) and zinc (Zn) [70]. However, the concentration threshold value for uranium hyperaccumulators is still undetermined. Several studies have reported that, the plant stem and leaf parts accumulate much less uranium than other heavy metals [71,72,73]. In this experiment, the bioconcentration factor PCF of Sudan grass roots was found to be 0.75 and the translocation factor TF was found to be 0.03, which was increased to 0.85 for PCF and 0.10 for TF after treatment with B6. Unlike other traditional hyperaccumulating plants with PCF greater than 1, Sudan grass has a PCF less than 1. However, there is no precisely defined uranium hyperaccumulating plant considering the specificity of uranium [71], and the short maturation cycle of Sudan grass leads to the possibility of increasing the uranium removal rate per unit of time or per unit of area of the plant by multiple sowing and harvesting [27], making it a promising potential plant for uranium enrichment.

Uranium primarily exists in soil in oxidized states, such as UO_2_^2+^ and U_3_O_8_ (note, U_3_O_8_ is typically not in a +2 oxidation state but rather an average of +6 across the molecule), and its behavior is influenced by factors like redox potential, pH, organic matter, and microorganisms [74,75]. Plants absorb U(IV) and U(VI) through epidermal root cells; however, the exact molecular mechanisms governing this uptake remain unclear. Most uranium ions, such as UO_2_^2+^, are taken up by roots via carriers or ion channels similar to those used for essential elements like calcium, iron, and magnesium [76]. In soil solutions, uranium predominantly occurs as UO_2_^2+^ and [UO_2_OH]^+^, and it can be absorbed by plants either through active transport processes that require energy or through passive diffusion. Although plants are capable of absorbing uranium from the soil, phytoremediation alone often has difficulty achieving the desired results due to a variety of factors such as soil properties and nutrient element availability [77]. The isolation, screening, and application of stress-tolerant PGPB for the improvement of plant productivity is important for meeting the nutrition requirement of plant growth and the enhancement of removal efficiency [68,69,78]. Therefore, we attempted to isolate some native PGPB with tolerance against heavy metal stress and multiple PGP capabilities from the mining site. The multiple-stress-tolerant *B. cereus* strain B6 was screened out and was used in the germination and pot experiments for enhancement of Sudan grass growth under contaminated soil. Phylogenetic analysis based on genome sequence showed that strain B6 is affiliated with the *Bacillus cereus* clade. The genome of B6 contains several genes related to stress resistance and growth promotion, such as nifU, glnA, and narK, which are involved in nitrogen fixation and nitrogen metabolism; afuABC and entABCE, which are involved in iron carrier synthesis and metabolism; and phoA, phoB, phnX, phoU, phoP, and phoR, which are involved in phosphate solubilization and transport. The in vitro experiments that followed confirmed these growth-promoting characteristics. *Bacillus* spp. show significant application advantages in bioremediation due to their unique survival mechanism: they were able to form dormant spores under extreme conditions, thus effectively resisting the stress of harsh environments [79,80]. *Bacillus cereus* was found to possess a variety of tolerance properties to heavy metals such as Cd, Pb, Cu, Zn. Here, we reported strain B6, which possess tolerance against uranium. Due to their ubiquity and close interaction with plants, *Bacillus cereus* were often considered as PGPB to promote many plant species such as soybean [81,82], maize [83], and lawn plants [27] and were also capable to promote plant growth effectively in stressful environmental conditions [84,85,86]. B. cereus strains can effectively mitigate the damage caused by abiotic stresses primarily through nutrition utilization, auxin production, increasing antioxidant activity, and changing the bioavailability of heavy metals [87,88,89,90]. In this study, *B. cereus* B6 has the properties of phosphorus solubilization, potassium solubilization, nitrogen fixation, siderophore production, organic acid synthesis, and protease and cellulase synthesis, which can convert these insoluble nutrients in the soils inverted into soluble forms, thus improving the effectiveness of NPK and others. Siderophores can not only acquire Fe^3+^ in the environment in a competitive manner, enabling plants to obtain the necessary iron nutrients [91,92], but are also able to chelate heavy metals so as to make them more easily absorbed by hyperaccumulating plants [93,94]. Microbial metabolites were one of the main sources of the low-molecular-weight organic acids (LMWOAs) in soils [95]. Organic acid not only increased the phosphorus availability in the rhizosphere [96] but also involved in metal detoxification, bioavailability, and accumulation [97,98].

Studies have shown that the application of plant growth-promoting Bacillus cereus strains can substantially improve various physiological and biochemical aspects of plants when they are subjected to stress [99,100]. These beneficial bacteria notably promote better germination, increased shoot and root lengths, and higher dry and fresh weights in plants. Additionally, they positively influence several biochemical indicators, including chlorophyll levels, MDA levels, glutathione (GSH), proline levels, and the overall antioxidant capacity. In this study, after inoculation treatment with strain B6, this inhibition effect on germination and growth was significantly alleviated, and the germination index, root length, and dry weight of Sudan grass were significantly higher than those uninoculated control groups under uranium stress. As uranium continues to accumulate in the plant, it leads to the production of excessive ROS, such as hydrogen peroxide (H_2_O_2_^−^) and superoxide (O_2_^−^), which triggers an imbalance between ROS generation and scavenging, and then causes cellular oxidative damage to reactive oxygen species (ROS) [71,101]. In this study, uranium toxicity reduced the activities of antioxidant enzymes and resulted in increased MDA in plants. It has been reported that inoculation with plant growth-promoting bacteria (PGPB) increases the activities of peroxidase (POD), superoxide dismutase (SOD), and catalase (CAT) while decreasing malondialdehyde (MDA) content in plants grown in heavy metal-contaminated soils [102,103]. Our results similarly show that inoculation with Bacillus cereus strain B6 enhances antioxidant activities and reduces MDA content compared to uranium-exposed plants. These findings are consistent with those reported by Akhtar et al. [64], who observed increased activities of CAT, SOD, and POD, along with decreased MDA content, in *Brassica nigra* (L.) grown in chromium-contaminated soil following inoculation with *B. cereus*. By enhancing the plant’s antioxidant system, strain B6 accelerates the removal rate of reactive oxygen species (ROS), alleviates oxidative stress caused by uranium exposure, and enhances the tolerance and adaptability of Sudan grass to uranium stress.

Similar to most reported cases, uranium was primarily retained in the roots of plants, with limited transfer to the aboveground parts [71,101,104]. It was found that the roots of Sudan grass were the main tissues of uranium accumulation in uranium-contaminated environments, and their uranium content was much higher than that of the aboveground parts. It was documented that PGPB increase HM accumulation in plants such as Cd in *Solanum nigrum* L. [105], Cr in *maize* [106], As in *Alnus* [107], etc. After treatment with strain B6, the uranium content of roots increased by 12%, and the uranium content of aboveground parts increased by 274%. It showed that B6 not only enhanced the uranium enrichment ability of Sudan grass roots but also promoted the transfer of uranium in the plants, which increased the accumulation of uranium in the whole plant. The capacity of plants to accumulate and absorb heavy metals (HMs) is largely contingent upon the bioavailable levels of these metals in the soil [108]. The introduction of Bacillus cereus can stimulate the mobilization of heavy metals in the rhizosphere, improving their bioavailability and enhancing their mobility. This, in turn, boosts the plants’ ability to absorb and accumulate heavy metals [26].

## 5. Conclusions

The native PGPR strain B6 was identified as Bacillus cereus. Strain B6 showed multiple heavy metal resistance and possessed plant growth-promoting properties such as phosphorus solubilization, iron carrier production, organic acids, protease and cellulase activities, etc., and has a good potential for uranium tolerance and growth promotion. The results of in vivo germination and potting tests of Sudan grass under uranium stress showed that strain B6 was able to alleviate the toxic effects of uranium stress on seed germination and the development of Sudan grass, significantly increase biomass production, and enhance the accumulation and transfer of uranium in the plant, thereby improving the restoration efficiency of Sudan grass. In conclusion, the results of this study suggest that the native PGPR Bacillus cereus B6 strain isolated in this study may be an effective biofertilizer for growing Sudan grass in uranium-contaminated soil. This study confirms the potential and prospect of the combined use of Sudan grass and Bacillus cereus for remediation of uranium-contaminated soil and provides a theoretical basis for the application of microorganisms combined with Sudan grass for remediation of uranium contamination of soil.

## Figures and Tables

**Table 1:** Physicochemical properties of soil for potting experiments.

Soil Parameters	Soil Samples
pH	5.25
Water, %	16.93
Organic, g·kg^−1^	59.42
TN, g·kg^−1^	2.15
Salt, g·kg^−1^	0.3
TP, g·(100 g)^−1^	0.024
TK, g·(100 g)^−1^	1.39
U, mg·kg^−1^	100

**Table 2:** Characterization of Bacillus cereus B6 genome and its comparison with other closely related Bacillus species.

Strain Characteristics	B6	*B. cereus ATCC 14579*	*B. thuringiensis ATCC 10792*	*B. albus N35-10-2*	*B. wiedmannii SR52*	*B. tropicus JMT105-2*	*B. proteolyticus TD42*
GeneBank No.	CP133649	NZ_CP034551.1	NZ_CM000753.1	CP128152.1	NZ_CP032365.1	CP115303.1	GCF_001884065.1
Size	5.7 M	5.4 M	6.2 M	5.8 M	5.3 M	5.5 M	5.9 M
G+C (%)	35.06	35.29	34.82	34.95	35.19	35.35	35.15
CDs	5909	5255	6243	5617	5255	5218	5749
ANI	/	97.16	96.5	91.34	91.48	91.79	89.69
dDDH	/	74	68.4	44.2	44.8	45.8	39.5

**Table 3:** Effects of uranium stress and *B. cereus* strain B6 inoculation on germination attributes of Sudan grass.

Treatment	Germinative Energy (%)	Germination Percentage (%)	Germination Index	Vital Index	Root Length (mm)	Shoot Length (mm)
T0	80.0 ± 0.0 ab	100 ± 0.0 a	40.2 ± 0.7 b	3107.2 ± 176.4 b	77.3 ± 2.9 b	66.5 ± 1.8 a
T1	90.0 ± 0.0 a	100 ± 0.0 a	44.1 ± 0.6 a	3962.3 ± 186.5 a	89.8 ± 3.1 a	67.8 ± 2.3 a
T2	83.3 ± 2.9 ab	90.0 ± 5.0 b	33.8 ± 1.3 c	1056.8 ± 20.0 d	31.3 ± 0.6 d	53.4 ± 1.4 b
T3	88.3 ± 2.9 a	95.0 ± 0.0 ab	38.3 ± 1.2 b	1629.0 ± 26.2 c	42.5 ± 1.7 c	55 ± 0.5 b
T4	73.3 ± 2.9 b	80.0 ± 5.0 c	26.1 ± 1.5 e	568.8 ± 65.0 f	21.7 ± 1.3 f	51.3 ± 4.6 b
T5	78.3 ± 7.7 b	90.0 ± 8.7 b	30.8 ± 1.9 d	850.6 ± 31.4 e	27.7 ± 0.9 e	52.2 ± 1.9 b

Abbreviations: T0 = 0 mg·L^−1^, T1 = 0 mg·L^−1^ + B6, T2 = U − 50 mg·L^−1^, T3 = U − 50 mg·L^−1^ + B6, T4 = U − 100 mg·L^−1^, T5 = U − 100 mg·L^−1^ + B6. Different letters indicate significant longitudinal difference in the column (*p* < 0.05).

**Table 4:** Effect of strain B6 on plant extraction and translocation of soil U.

Treatment	Soil Uranium Concentration	Root Uranium Concentration	Shoot Uranium Concentration	PCF	TF
unit	mg·kg^−1^	mg·kg^−1^	mg·kg^−1^		
CK	100	74.70 ± 0.92	2.281 ± 0.29	0.75 ± 0.009	0.03 ± 0.004
B6	100	84.91 ± 2.68 *	8.56 ± 0.60 *	0.85 ± 0.028 *	0.10 ± 0.004 *

* *p* < 0.05.

## Data Availability

The original contributions presented in this study are included in the article/Supplementary Material. Further inquiries can be directed to the corresponding author(s).

## Supplementary Materials

The following supporting information can be downloaded at: https://www.mdpi.com/article/10.3390/biology14010058/s1, Figure S1: Growth curves of strain B6 in NB at different heavy metal concentrations (A: Cadmium, Cd; B: Chromium, Cr; C: Lead, Pb; D: Manganese, Mn; E: Zinc, Zn); Figure S2: Effect of B6 on seed germination of Sorghum sudanense. Table S1: The 14 copies of 16 s rRNA genes predicted in the chromosome of strain B6, and the top-hit results by blasting against EzTaxon database.; Table S2: Metals resistance and plant-growth promoting related genes identified in the sequenced whole genomes of the strain B6.
