# Supplementary material for: Bioprospecting of a Native Plant Growth-Promoting Bacterium Bacillus cereus B6 for Enhancing Uranium Accumulation by Sudan Grass (Sorghum sudanense (Piper) Stapf)
Source: Biology (Basel). 2025 Jan 13;14(1):58. doi: 10.3390/biology14010058 (PMC11762556; doi:10.3390/biology14010058)
Supplement: Supplementary file 1 [file biology-14-00058-s001.zip › biology-3378316-supplementary.pdf]

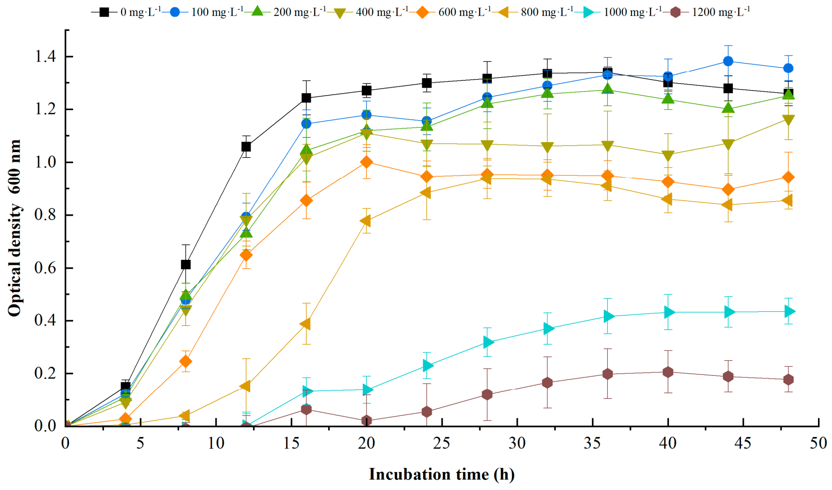
0 mg·L-1
100 mg·L-1
200 mg·L-1
400 mg·L-1
600 mg·L-1
800 mg·L-1
1000 mg·L-1
1200 mg·L-1
Optical density 600 nm
Incubation time (h)

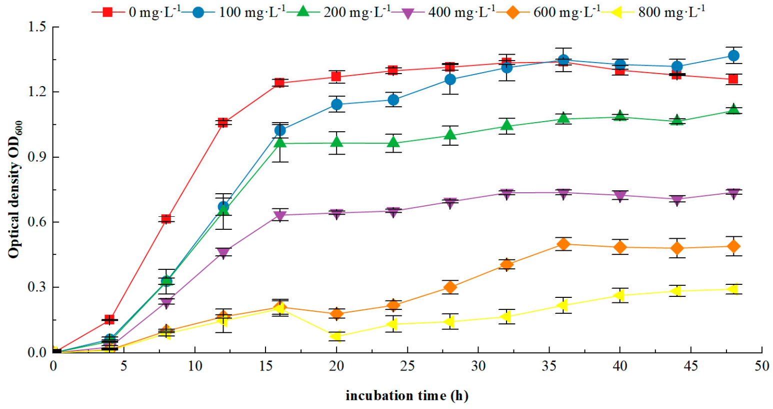
0 mg·L-1
100 mg·L-1
200 mg·L-1
400 mg·L-1
600 mg·L-1
800 mg·L-1
Optical density OD600
incubation time (h)

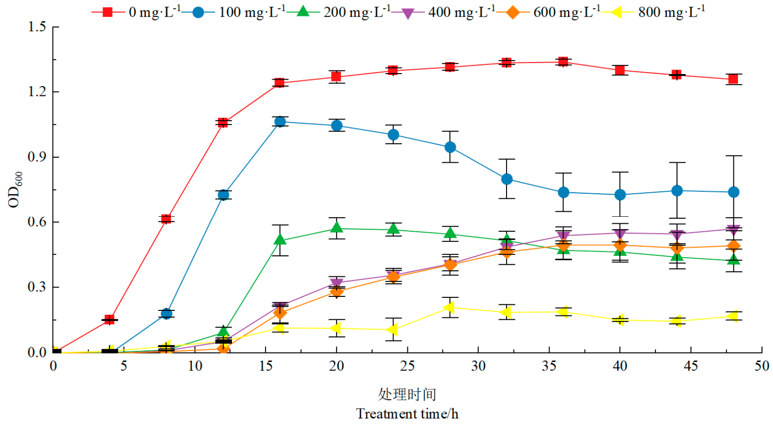
0 mg·L-1
100 mg·L-1
200 mg·L-1
400 mg·L-1
600 mg·L-1
800 mg·L-1
OD600
处理时间
Treatment time/h

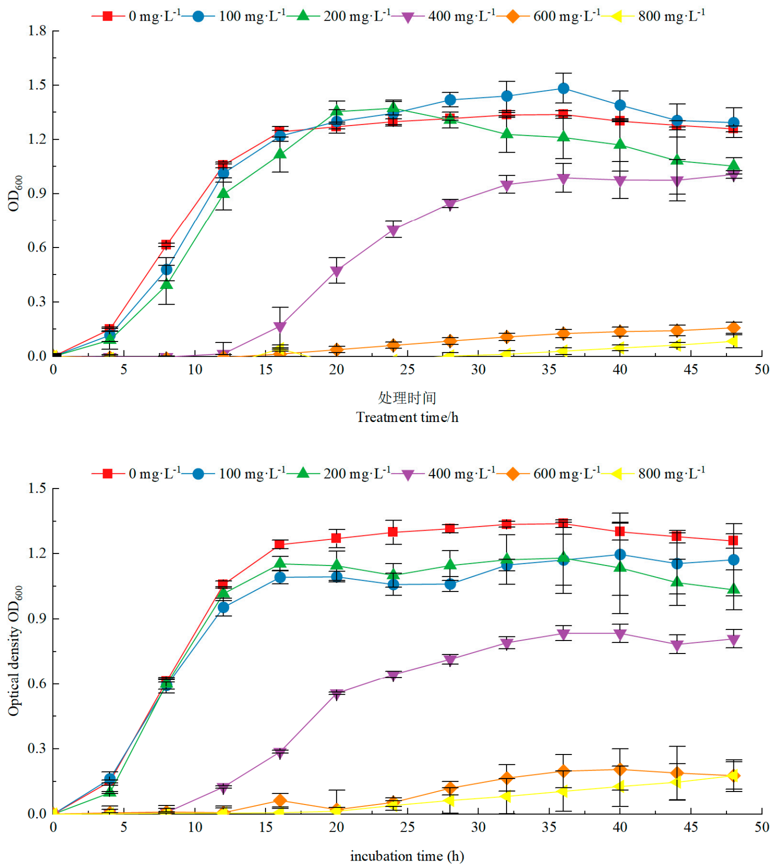

Figure S1 Growth curves of strain B6 in NB at different heavy metal concentrations (A: Cadmium, Cd; B: Chromium, Cr; C:Lead, Pb; D: Manganese, Mn; E:Zinc, Zn)

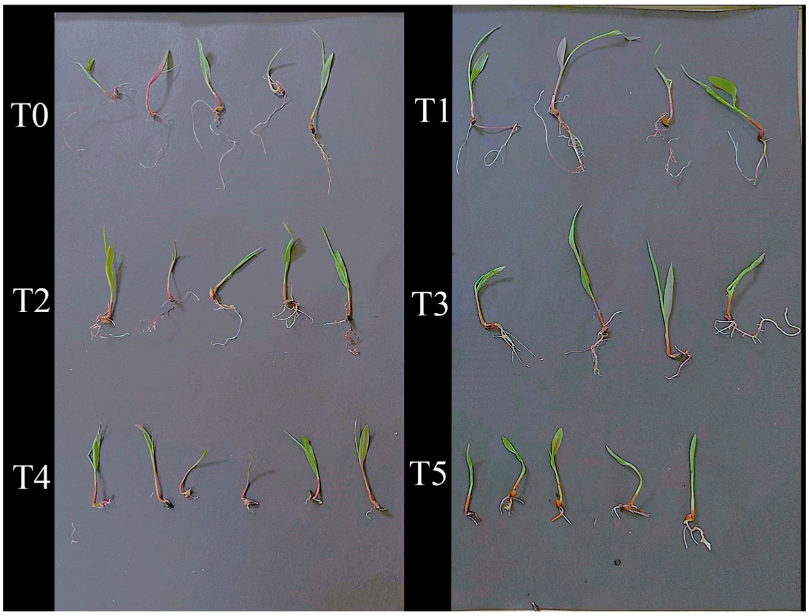

Figure S2 Effect of B6 on seed germination of Sorghum sudanense.

T0 = 0 mg·L^{-1}, T1 = 0 mg·L^{-1} + B6, T2 = U − 50 mg·L^{-1}, T3 = U − 50 mg·L^{-1} + B6, T4 = U − 100 mg·L^{-1}, T5 = U −

100 mg·L^{-1} + B6.

Supplementary Table S1

The 14 copies of 16 s rRNA genes predicted in the chromosome of strain B6, and the top-hit results by blasting against EzTaxon database.

16s rRNA	location From -to	Top-hit taxon	Top-hit strain	Similarity (%)	Completeness (%)
1	720384-721926	*Bacillus proteolyticus*	TD42	99.93	100
2	4655756-4657294	*Bacillus proteolyticus*	TD42	99.93	100
3	513342-514881	*Bacillus proteolyticus*	TD42	99.93	100
4	267606-269144	*Bacillus cereus*	ATCC 14579	100	100
5	278024-279568	*Bacillus cereus*	ATCC 14579	100	100
6	260340-261879	*Bacillus cereus*	ATCC 14579	100	100
7	28660-30198	*Bacillus cereus*	ATCC 14579	100	100
8	150871-152410	*Bacillus albus*	N35-10-2	99.93	100
9	239768-241306	*Bacillus albus*	N35-10-2	100	100
10	87652-89193	*Bacillus albus*	N35-10-2	99.93	100
11	81943-83482	*Bacillus albus*	N35-10-2	100	100
12	291346-292885	*Bacillus albus*	N35-10-2	100	100
13	298505-300043	*Bacillus albus*	N35-10-2	100	100
14	8916-10456	*Bacillus albus*	N35-10-2	99.86	100

Top-hit taxonomy: Bacteria; Firmicutes; Bacilli; Bacillales; Bacillaceae; Bacillus

Supplementary Table S2

Metals resistance and plant-growth promoting related genes identified in the sequenced whole genomes of the strain B6

PGP Activities	Star	Stop	Length(bp)	Gene name	Gene Annotation
Nitrogen metabolism	3542688	3541354	1335	glnA, GLUL	glutamine synthetase
	620273	618390	1884	norD	Nitric oxide reductase activation protein NorD
	621170	620277	894	norQ	nitric oxide reductase NorQ protein
	1998910	1998593	318	nirD	nitrite reductase (NADH) small subunit
	2001331	1998926	2406	nirB	nitrite reductase (NADH) large subunit
Iron sequestration	611631	610678	954	afuA, fbpA	iron(III) transport system substrate-binding protein
	613876	614697	822	afuC, fbpC	iron(III) transport system ATP-binding protein
	611804	612808	1005	afuB, fbpB	iron(III) transport system permease protein
	2165273	2166058	786	entA	2,3-dihydro-2,3-dihydroxybenzoate dehydrogenase of siderophore biosynthesis
	2166084	2167283	1200	entC	Isochorismate synthase of siderophore biosynthesis
	2167296	2168912	1617	entE	2,3-dihydroxybenzoate-AMP ligase of siderophore biosynthesis
	2168937	2169830	894	entB	Isochorismatase of siderophore biosynthesis
Phosphate solubilization	2826794	2825121	1674	phoA,	Alkaline phosphatase
	4151990	4150605	1386	phoB	Alkaline phosphatase
	3840784	3839246	1539	ppx-gppA	Exopolyphosphatase

	1316208	1317002	795	phnX	Phosphonoacetaldehyde hydrolase
	1317018	1318115	1098	phnW	2-aminoethyl phosphonate:pyruvate aminotransferase
	2670715	2669786	930	ppaC	Manganese-dependent inorganic pyrophosphatase
	4079908	4079252	657	phoU	Phosphate transport system regulatory protein PhoU
	4114182	4113223	960	phoH	Phosphate starvation-inducible protein PhoH, predicted ATPase
	4372836	4372117	720	phoP	Alkaline phosphatase synthesis transcriptional regulatory protein PhoP
	4372124	4370361	1764	phoR	Phosphate regulon sensor protein PhoR
Heavy metal resistance	4931996	4933177	1182	chra	chromate transporter
	588362	588739	378	CzcD	cobalt-zinc-cadmium efflux system protein
	1899107	1900048	942	corA	magnesium transporter
	1776516	1778150	1635	CopC or CopD	Copper resistance protein
	2913238	2912561	678	CutC	Cytoplasmic copper homeostasis protein
	820572	820931	360	litR	Transcriptional regulator, MerR family
	1458680	1459555	876	cueR	
	1566213	1567058	846	glnR	
	1652611	1652231	381	yfmP	
	1773087	1772122	966	ACR3	Arsenical-resistance protein

2995064	2994024	1041	arsB	
2993998	2993594	405	ARSC2	Arsenate reductase
3166633	3166238	396	arsC	
